# Screening of Gene Expression Markers for Corona Virus Disease 2019 Through Boruta_MCFS Feature Selection

**DOI:** 10.3389/fpubh.2022.901602

**Published:** 2022-06-22

**Authors:** Yanbao Sun, Qi Zhang, Qi Yang, Ming Yao, Fang Xu, Wenyu Chen

**Affiliations:** ^1^Department of Radiology, Affiliated Hospital of Jiaxing University, Jiaxing, China; ^2^Department of Respiration in Affiliated Hospital of Jiaxing University/The First Hospital of Jiaxing, Jiaxing, China; ^3^Center for Pain Medicine in Affiliated Hospital of Jiaxing University/The First Hospital of Jiaxing, Jiaxing, China; ^4^The Xiuzhou Kang'an Hospital of Jiaxing, Jiaxing, China

**Keywords:** COVID-19, feature selection, random forest classifier, gene expression markers, bioinformatics

## Abstract

Since the first report of SARS-CoV-2 virus in Wuhan, China in December 2019, a global outbreak of Corona Virus Disease 2019 (COVID-19) pandemic has been aroused. In the prevention of this disease, accurate diagnosis of COVID-19 is the center of the problem. However, due to the limitation of detection technology, the test results are impossible to be totally free from pseudo-positive or -negative. Improving the precision of the test results asks for the identification of more biomarkers for COVID-19. On the basis of the expression data of COVID-19 positive and negative samples, we first screened the feature genes through ReliefF, minimal-redundancy-maximum-relevancy, and Boruta_MCFS methods. Thereafter, 36 optimal feature genes were selected through incremental feature selection method based on the random forest classifier, and the enriched biological functions and signaling pathways were revealed by Gene Ontology and Kyoto Encyclopedia of Genes and Genomes. Also, protein-protein interaction network analysis was performed on these feature genes, and the enriched biological functions and signaling pathways of main submodules were analyzed. In addition, whether these 36 feature genes could effectively distinguish positive samples from the negative ones was verified by dimensionality reduction analysis. According to the results, we inferred that the 36 feature genes selected via Boruta_MCFS could be deemed as biomarkers in COVID-19.

## Introduction

Since the first report of severe acute respiratory syndrome coronavirus type 2 (SARS-CoV-2) in Wuhan in December 2019, the pandemic of the Coronavirus Disease 2019 (COVID-19) has swept the whole world. As of March 1, 2021, according to data published by the World Health Organization, SARS-CoV-2 has caused 113,820,168 infected cases and 2,527,891 deaths (https://www.who.int/en/). SARS-CoV-2 infection is mainly characterized by high viral load and high infectivity in patients at (or before) the onset of COVID-19 symptoms, while a proportion of infected individuals are asymptomatic (1–4). Therefore, the precise diagnosis for COVID-19 is of great importance. Presently, assistance of COVID-19 diagnosis broadly covers the following ways: detecting the viral RNA through qPCR; detecting antigens or corresponding antibodies in serum by colloidal gold or chemiluminescence; lung images via computed tomography (CT). However, none of these methods could avoid missed diagnosis or misdiagnosis of COVID-19 (5). To improve the diagnostic precision of COVID-19, it is urgent to discover new biomarkers.

ReliefF, minimal-redundancy-maximum-relevancy (mRMR) and BorutaMCFS were adopted to screen feature genes from expression data. RelieF is an algorithmic process for assessing the weight ratio percentage of multiple attributes in a system, and is often used in practical applications to preprocess data to obtain a feature subset (6). mRMR is an algorithm that measures the relevance and redundancy of features, and selects the one with maximum relevance and minimum redundancy. This approach focuses on preprocessing data to improve prediction accuracy (7). Tao Li et al. proved that ReliefF, mRMR, and the combination of the two could select feature genes from different tumor samples based on gene expression data. However, after being validated by support vector machine (SVM) and Naïve Bayes Classifier (NB), the optimal feature genes were selected through the combination of ReliefF and Mrmr (6). Boruta is a random forest-based screening approach. It iteratively removes features that have been proven to be less correlated with random probes, which in turn reduces signal noise (8). Degenhardtet al. (9) used Boruta to screen feature genes of breast cancer patients and classified ER-positive and -negative samples by a random forest classifier, which was indicated to have a stable classification effect. However, the importance of the features identified through this approach could not be determined. Hence, we further performed MCFS feature selection based on Boruta. MCFS is a feature selection method based on random sampling and constructing multiple decision trees (10). In a study by Yu-DongCai et al. (10) MCFS was adopted to preprocess peptide chain profiling data, which in turn led to the selection of peptide chains that could effectively classify different cancers. The combination of Boruta and MCFS was adopted in this study.

In this study, we selected 36 effective feature genes as biomarkers for COVID-19 on the basis of their expression data in COVID-19 positive and negative samples with the combination of Boruta and MCFS methods. Through enrichment analysis, literature review, and principal component analysis (PCA), these feature genes were evaluated for their qualification as COVID-19 biomarkers. Based on the analysis results, we concluded that the identified 36 feature genes were expected to be novel biomarkers for COVID-19.

## Materials and Methods

### Study Design and Acquirement of Expression Data of Genes and MRNAs Related to COVID-19

**Figure 1:**
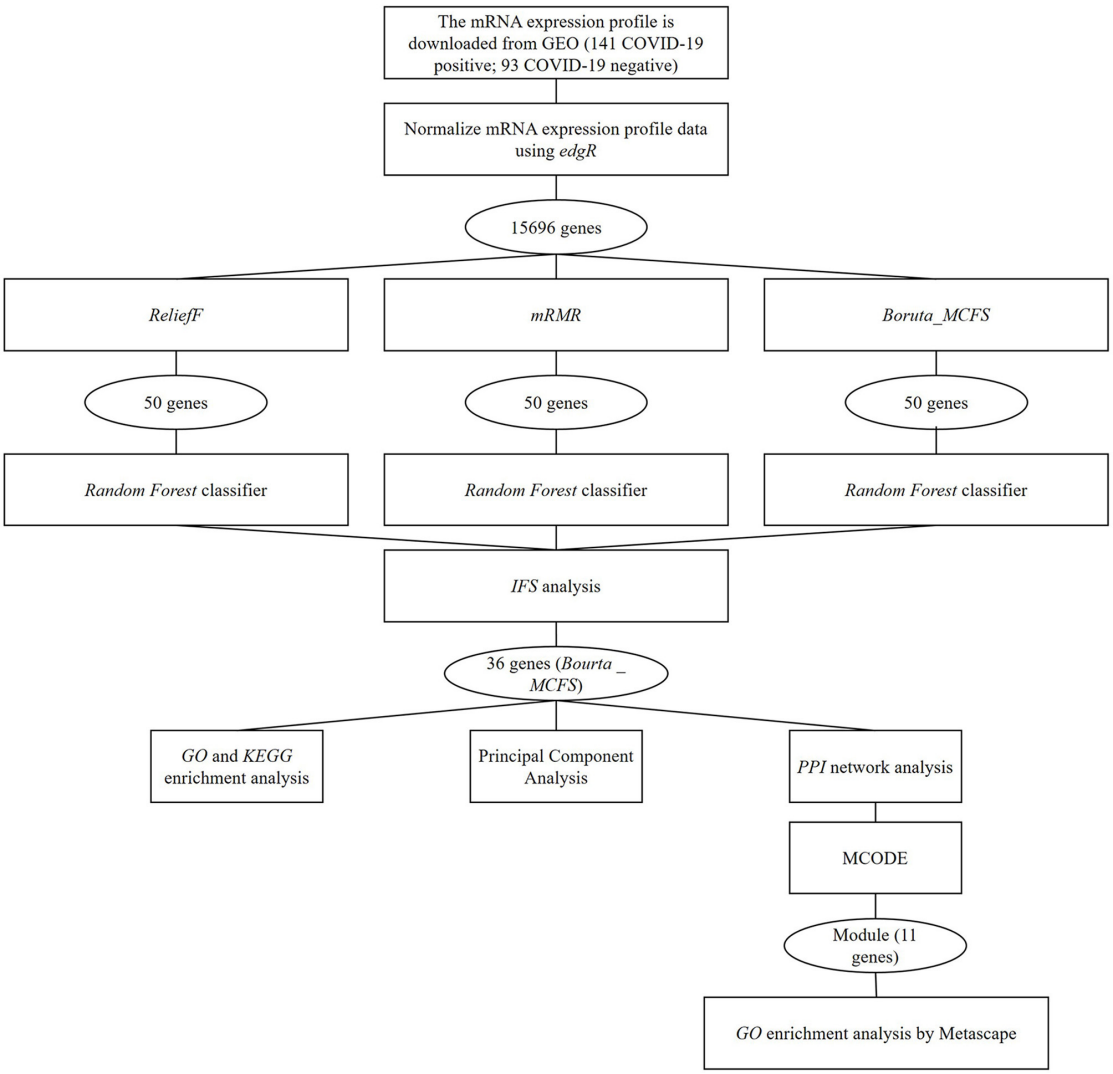
The flowchart of the study.

COVID-19-related data were acquired by following steps mentioned in literature (11). Specifically, the gene expression data contained upper respiratory tract mRNA expression data (GSE156063) from 93 COVID-19 patients with acute respiratory disease and 141 uninfected patients with acute respiratory disease. The expression data were downloaded from the Gene Expression Omnibus (GEO) database (https://www.ncbi.nlm.nih.gov/geo/). Clinical characteristics of 234 samples were manifested in Supplementary Table 1. The mRNA expression data were obtained through RNA metagenomic sequencing with GPL24676 Illumina NovaSeq 6000 (Homosapiens). In the expression matrix, genes with mean values <1 and maximum values >5 were removed. The remaining data were subsequently normalized by the edgR package (12). Based on the dataset, the flowchart of the study is displayed in Figure 1.

### Feature Genes Screening

The data were classified by ReliefF (13) and the feature genes were ranked by the mRMR method (11). Algorithm 1 is a novel algorithm based on Relief (14). Relief is an algorithm that assigns features different weights based on the relevance of each feature and category, and features with weights less than a specific threshold will be removed. Since Relief can only process two categories of data, ReliefF, which can process multiple categories of sample data, was later developed based on Relief algorithm. This algorithm is used to deal with regression problems where the targets are continuous values. The processes of ReliefF algorithm were as follows: Sample R_i_ is randomly taken from the dataset W[A] each time, and then k nearest neighbor samples H_j_ (nearest hits) are found from the set of Ri similar samples, while k nearest neighbor samples M_j_ (nearest misses) are found from the set of R_i_ non-similar samples. The weight of each feature is updated according to the R_i_, H_j_, M_j_ values, and ranked according to each feature weight.

**Algorithm 1:** ReliefF.

**Input: for each training instance a vector of attribute values and the class value Output: the vector W of estimations of the qualities of attributes 1. set all weights W[A]: = 0.0; 2. for i: = 1 to m do begin 3. randomly select an instance Ri; 4. find k nearest hits Hj; 5. for each class C ≠ class(Ri) do 6. from class C find k nearest misses Mj(C); 7. for A: = 1 to a do 8. $\text{W}\left[\text{A}\right]:=\text{W}\left[A\right]-\overset{k}{\underset{j=1}{\sum }}diff\left(A,\;Ri,\;Hj\right)/\left(m\;k\right)+$ 9.$\sum_{\text{C}\neq \text{class}(\text{Ri})}\frac{\left[\frac{p\left(C\right)}{\text{1}-\text{p}(\text{class}\left(Ri\right)}\sum_{j=1}^{k}diff\left(\text{A},\text{ Ri},\text{ Mj}\left(\text{C}\right)\right)\;\right]}{m.k};$ 10. end;**


The mRMR is an algorithm (7) that measures the relevance and redundancy of features and picks those with the maximum relevance (Max-Relevance) and the minimum redundancy (Minimal-Redundancy). Max-Relevance was calculated with the following formula:


$$\begin{array}{l} maxD\left(S,c\right),\;D=\frac{1}{\left|S\right|}\underset{x_{i}\in S}{\sum }I\left(x_{i};c\right) \end{array}$$

Features selected on the basis of Max-Relevance can be redundant and have large interdependencies. Therefore, removing one feature from these mutually highly dependent features did not hugely change the classification results. To select independent features, Minimal-Redundancy was introduced:


$$\begin{array}{l} \min R\left(S\right),R\;=\;\frac{1}{|S{|}^{2}}\underset{x_{i},x_{j}\in S}{\sum }I\left(x_{i},x_{j}\right) \end{array}$$

In the above formula, S represents the feature set, x represents the feature, and c represents the classification. The algorithm mRMR combined Max-relevance and Minimal-Redundancy and is defined as:


$$\begin{array}{l} max\Phi \left(D,R\right),\;\Phi =D-R \end{array}$$

Boruta is a random forest-based screening approach. It iteratively removes features that have been shown to have low correlation with random probes, which in turn reduces signal noise (15). The algorithm for Boruta is listed below:

1. Enlarge the information system by adding sample data.
2. Disrupt the added attributes.
3. Run random forest classifier in the expanded information system, where Z scores were calculated.
4. Find the maximum Z-score in the shadow attribute (MZSA), and then assign a hit to each feature with scores higher than MZSA.
5. Perform a two-sided test equivalent to MZSA on attributes with undetermined significance.
6. Remove features significantly less important than MZSA from the information system.
7. Delete all the MZSA.
8. Repeat the algorithm until importance was assigned to all attributes.

However, the importance of features selected by this method could not be determined. Therefore, we further performed MCFS to select features based on Boruta results.

MCFS builds multiple decision trees based on random sampling in multiple characteristics and then infers relative importance (RI) of each feature through the repeated bootstrap tests (16). The MCFS algorithm is defined as:


$$\begin{array}{l} RI_{g}=\overset{p\times t}{\underset{\tau =1}{\sum }}{\left(wA_{CC}\right)}^{u}IG\left(ng\left(\tau \right)\right){\left(\frac{no.in\;ng\left(\tau \right)}{no.in\;\tau }\right)}^{v} \end{array}$$

In the formula, *wA*_*CC*_ was the weighted accuracy; *ng*(τ) was the node of the characteristic in the decision tree; the information acquisition of *ng*(τ) was expressed as *IG*(*ng*(τ)); *no*.*in ng*(τ) represents the number of training samples in *ng*(τ); u and v represent different weight factors, whose default value was 1.

Feature genes were screened through ReliefF, mRMR, and Boruta_MCFS. The ReliefF algorithm was based on the python package “sklearn.” The mRMR algorithm-related program (http://home.penglab.com/proj/mRMR/) was downloaded, by which the features were ranked. The Boruta feature selection method was constructed on the basis of the python package “Borutapy” for removing less correlated feature genes. Subsequently, the MCFS feature selection method was constructed based on the python package “skfeature” (17) to further identify important feature genes.

### The Construction of Classifier and the Selection of Optimal Feature Genes

Three sets of top 50 feature genes were selected by the three feature selection methods. Thereafter, we set up a classifier to filter optimal feature genes by methods described previously (18). A random forest classifier was constructed based on the python package “skfeature” (17). Owing to the unbalanced samples, model training was conducted on the basis of python package “imblearn” and upsampling method. An incremental feature selection (IFS) curve (19) was drawn based on the Matthews correlation coefficient (MCC) obtained based on the 10-fold cross-validation of random forest classification. MCC is the Pearson correlation coefficient of the actual and predicted values calculated by the confusion matrix method. MCC values range from −1 to +1, with values approaching +1 indicating a more precise prediction, values approaching 0 indicating the prediction is not better than random one, and values approaching −1 indicating the opposite relationship between predicted and actual observations (20). The feature gene selection method with the highest MCC value in the IFS curve and the corresponding top feature genes were selected as feature genes with good prediction performance.

### Enrichment Analyses

Optimal feature genes were subjected to pathway enrichment analyses by Gene Ontology (GO) and Kyoto Encyclopedia of Genes and Genomes (KEGG) (11). GO and KEGG enrichment analyses were performed by adopting the R package “clusterProfiler” (21) (*p*Value < 0.05, qValue < 0.05). GO enrichment analysis revealed the biological process (BP) and molecular function (MF) were mainly enriched by the feature genes. KEGG unveiled the relevant signaling pathways with major enrichment of feature genes. The protein-protein interaction (PPI) network subset analysis was carried out at Metascape (http://metascape.org/) (*p*Value <0 .05) for GO enrichment analysis (http://metascape.org/).

### PPI Network Analysis

Interactions between proteins were analyzed by PPI networks by ways described before (22). Through String database (https://www.string-db.org/), a PPI network analysis (with default parameters) was performed on the feature genes (with all parameters as default), major PPI network subsets were selected by using MCODE in Cytoscape. Meanwhile, we performed a topological analysis of the PPI network. GO enrichment analysis was performed on the subsets selected by Metascape.

### Principal Component Analysis (PCA)

Validity of the feature genes was verified by PCA method according to a previous report (11). PCA is a dimensionality reduction analysis for high latitude data. The R package “FactoMineR” was adopted to extract the first and second principal components of optimal feature genes (23). Through the dimensionality reduction analysis of high-latitude feature genes, the expression profile dataset is mapped to two dimensions to obtain sample scatter plots with different distances.

## Results

### Different Feature Selection Methods Including ReliefF, MRMR, and Boruta_MCFS Were Compared

**Figure 2:**
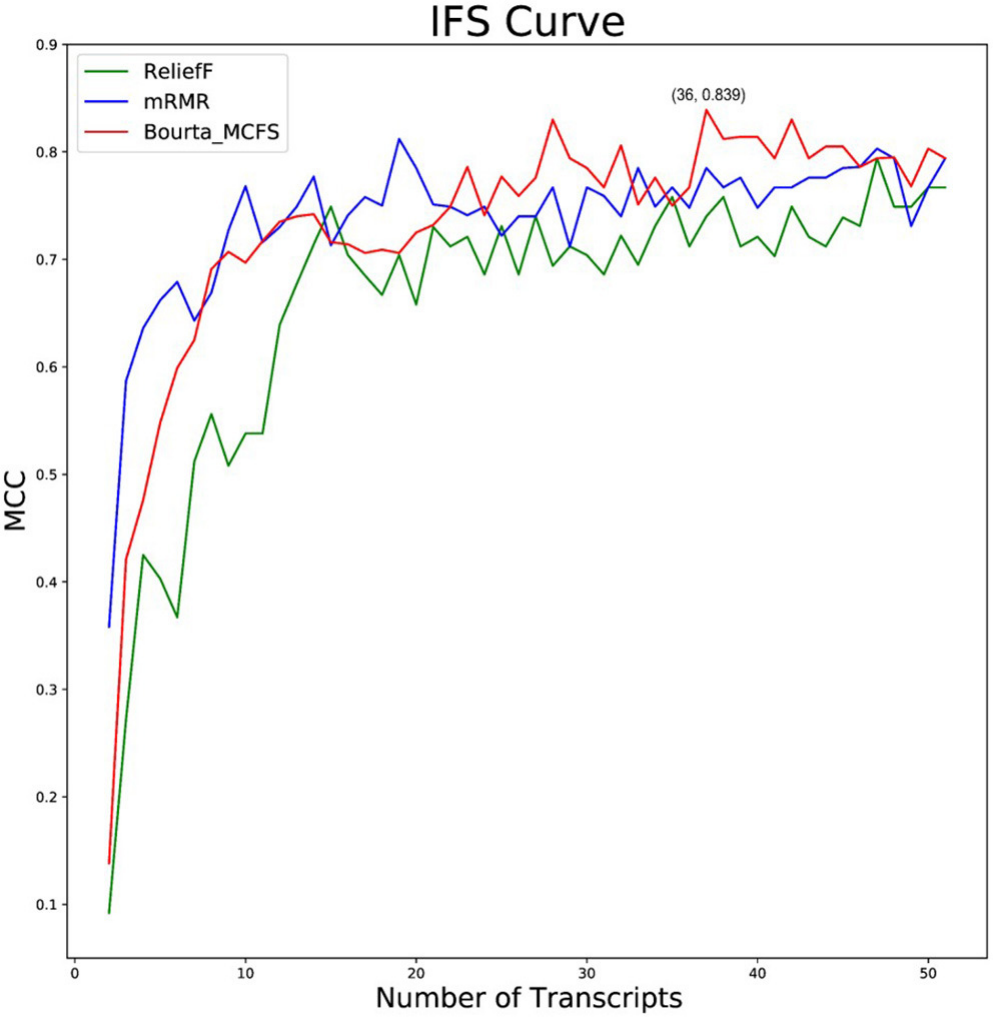
Different feature selection methods including ReliefF, mRMR, and Boruta_MCFS were compared. The IFS curves of ReliefF, mRMR, and Boruta_MCFS methods based on the random forest classifier. The abscissa represents the number of feature genes, and the ordinate represents the MCC value.

Based on the 15,696 genes obtained, we further used the ReliefF, mRMR, and BorutaMCFS algorithms for feature gene selection after data normalization by the package “edgeR.” We selected the top 50 feature genes using the above 3 methods (Supplementary Table 2). The obtained feature genes were classified through the random forest classifier and then subjected to the 10-fold cross-validation. Optimal feature gene set checked by IFS curve was used as biomarkers for COVID-19. The IFS curve revealed the highest MCC value, with a sensitivity of 0.892, a specificity of 0.923 and MCC of 0.839 (Figure 2) in the random forest classification model constructed with the 36 top feature genes (Table 1) selected by the Boruta_MCFS method.

**Table 1:** 36 feature genes screened by Boruta_MCFS feature selection method.

**Boruta_MCFS**			
PLVAP	SIGLEC1	SERPING1	IFIT5
TRO	IFI6	CXCL10	ATM
TMEM126A	LGR6	MED9	PTAFR
RTP4	PBDC1	LAG3	RILPL2
NOC3L	PADI2	SCN2A	PRMT7
ICAM4	ISG15	USP18	CDC42EP3
CXCL11	HERC5	OAS3	COPS5
BST2	HRASLS2	DDX58	PPARD
DSC2	NDUFB9	NPFFR1	IFI44

### Enrichment Analyses

**Figure 3:**
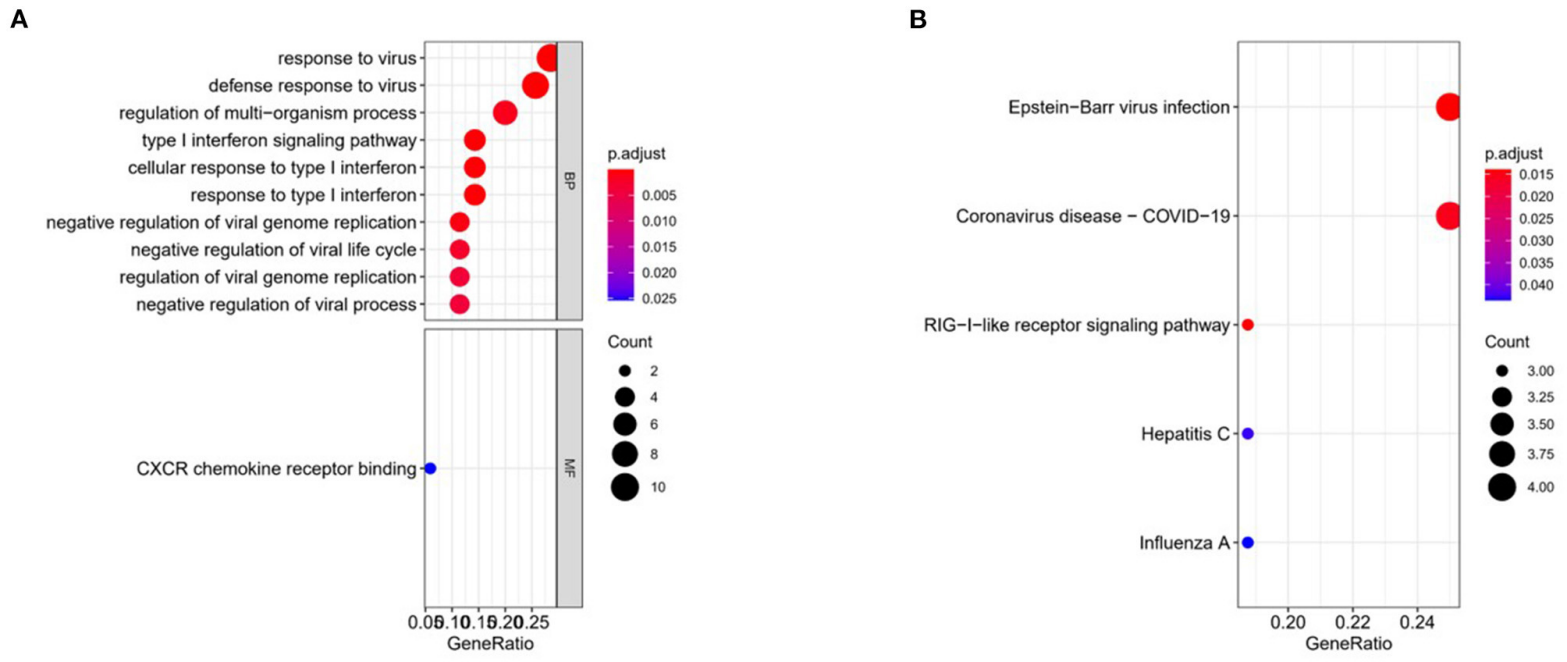
Functional enrichment analyses. **(A)** GO was performed on 36 feature genes. **(B)** KEGG was performed on 36 feature genes.

In order to explore the biological functions and signaling pathways involved by the feature genes, we performed GO and KEGG enrichment analyses on these 36 feature genes. These genes were mainly enriched in biological functions including response to virus, defense response to virus, regulation of multi-organism process, type I interferon signaling pathway, and negative regulation of viral genome replication (Figure 3A). KEGG revealed that these genes were largely enriched in signaling pathways including Epstein-Barr virus infection and Coronavirus-COVID-19 (Figure 3B). Besides, it could be predicted from the results that the signaling pathway of coronavirus infection may be similar to that of Epstein-Barr virus. Also, this assumption has also been pointed out in previous works through proteomic and transcriptomic analyses (24).

### PPI Network Analysis

**Figure 4:**
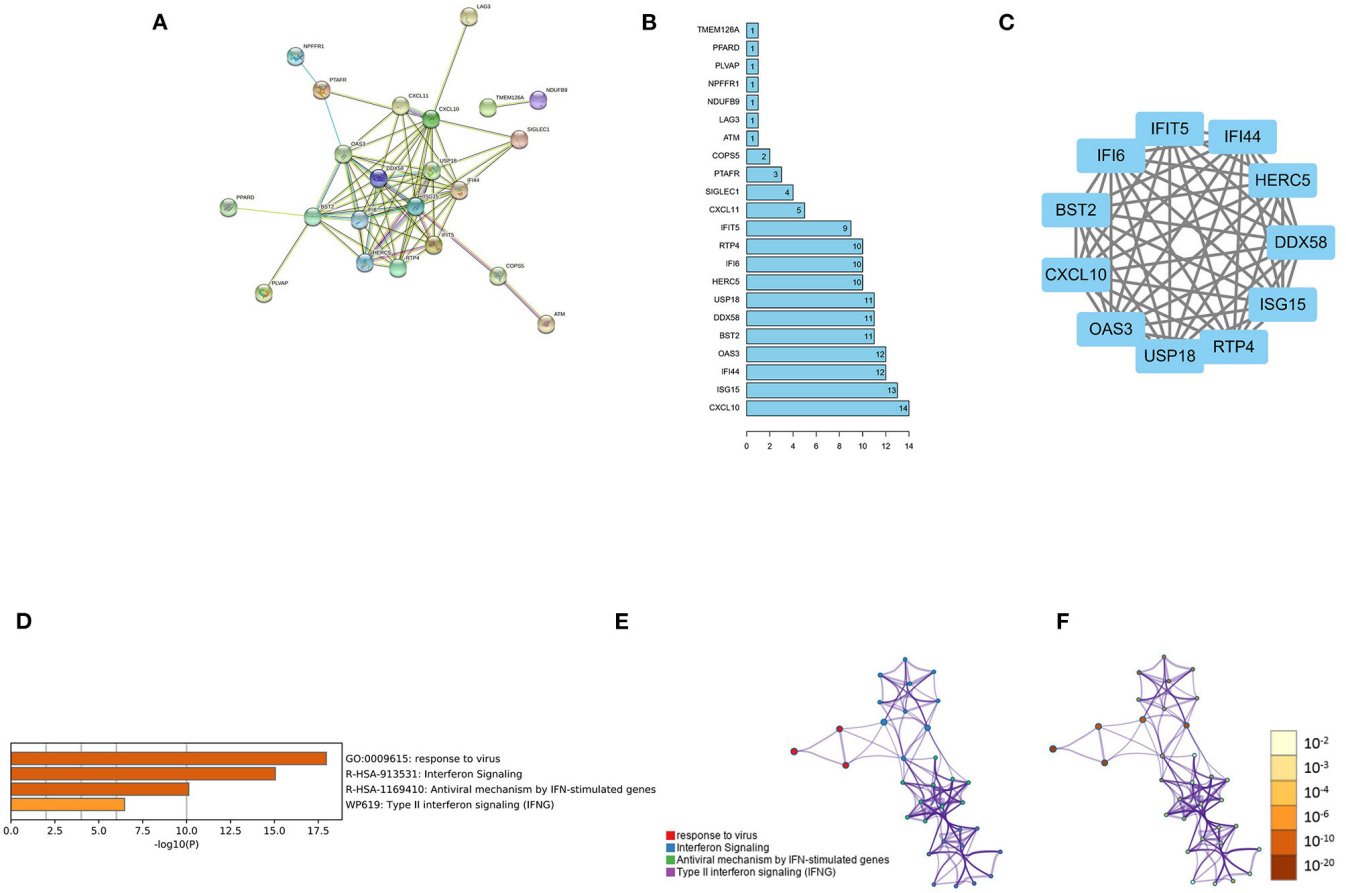
PPI network analysis. **(A)** The PPI network based on 36 feature genes. **(B)** The number of nodes of the 22 feature genes in the PPI network. **(C)** Major subsets identified using MCODE; **(D)** The heat map of the enriched module (the smaller the *p*-value, the darker the color). **(E)** The network of the enriched module (the same cluster with the same color). **(F)** Enriched module network (the smaller the *p*-value, the darker the color).

To further validate the relationship of the 36 feature genes, we performed PPI network analysis through the String database, where 73 interactions and 22 nodes were contained in the network (Figure 4A). Eleven out of 22 feature genes in the PPI network had more nodes: CXCL10, ISG15, IFI44, OAS3, BST2, DDX58, USP18, HERC5, IFI6, RTP4, and IFIT5 (Figure 4B). Then, based on the PPI network, the largest subset containing 11 nodes was selected through MCODE (Figure 4C). GO functional enrichment analysis was performed on the main subset on the Metascape website (Figures 4D–F). These genes were mainly enriched in biological functions including response to virus, interferon signaling, antiviral mechanism by IFN-stimulated genes, and type II interferon signaling.

### PCA

**Figure 5:**
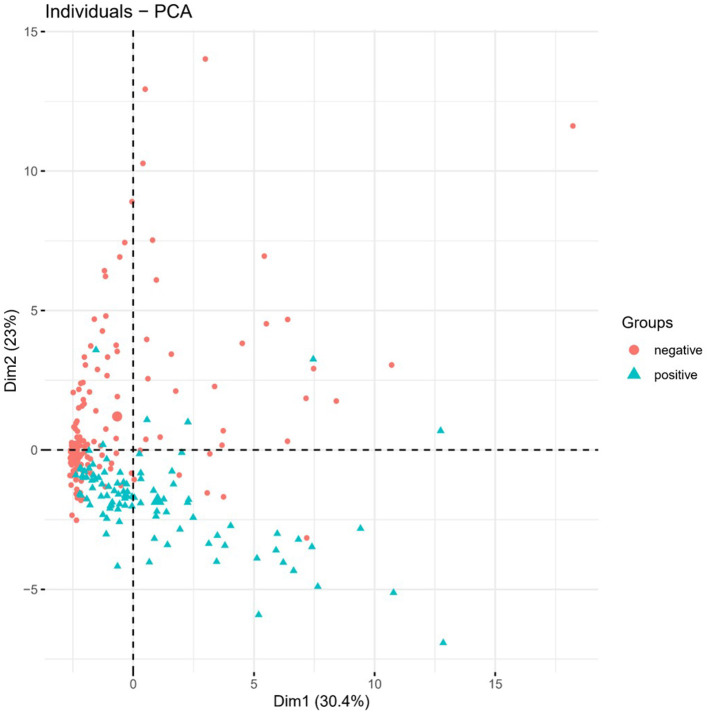
PCA. PCA was performed on the 36 feature genes. Green represents positive samples and red represents negative ones.

In order to verify whether the above 36 feature genes could effectively distinguish the positive cases from the negative ones, we performed PCA on these genes (Figure 5). The results suggested that positive and negative COVID-19 samples could be separated in PC1 and PC2. Therefore, we inferred that the above 36 feature genes could be used to judge whether the sample was COVID-19 positive or negative.

## Discussion

In this study, in order to find new biomarkers for COVID-19, we screened effective feature genes based on the expression data of COVID-19 positive and negative samples. Enrichment analysis, literature review, and PCA were performed to verify whether these feature genes could be COVID-19 biomarkers. First, 3 methods of ReliefF, mRMR, and Boruta_MCFS were adopted to screen feature genes from the expression data. Then, the optimal feature genes were confirmed by the random forest classifier based on IFS. Compared with ReliefF and mRMR, Boruta_MCFS can screen feature genes that are more reliable. We performed GO and KEGG enrichment analyses on the optimal feature genes and found that these genes were mainly enriched in biological functions and signaling pathways relating to SARS-CoV-2 and antiviral functions, as well as immune regulation. At the same time, PPI network analysis was also performed on the feature genes to confirm the main subsets in the network. GO and KEGG were performed on the subsets on the Metascape website. The results revealed that the main subset was enriched in the above-mentioned biological functions and signaling pathways. Finally, the PCA analysis verified that COVID-19 positive and negative samples could be distinguished by PC1 and PC2 based on the selected feature genes. Combining the results of all bioinformatics analyses, a COVID-19 classifier based on 36 feature genes was built. At the same time, we inferred that these 36 feature genes were expected to be novel biomarkers for COVID-19.

Based on the 36 selected feature genes, we performed further analysis in combination with literature review and found 13 genes (CXCL11, BST2, CXCL10, MED9, LAG3, USP18, OAS3, DDX58, IFI6, PADI2, ISG15, PTAFR, IFI44) were reported in articles relating to SARS-CoV-2 (25–35). According to the study reports, CXCL10, CXCL11, LAG3, OAS3, PADI2 and other genes are significantly upregulated in the blood or lung tissue in patients with severe COVID-19 (25, 27, 29, 31, 33, 36). The expression of BST2 and DDX58 is associated with antiviral functions (26, 30, 37). In addition, LAG3 and USP18 are involved in inhibiting the cytotoxicity of CD8+T cells and inhibiting IFN-I signaling pathway, respectively (29, 38, 39). Some existing studies also demonstrated that some genes are associated with COVID-19.

The functional enrichment analysis of 36 feature genes showed that these genes were mainly enriched in functions and pathways relating to SARS-CoV-2 and antivirus, as well as immune regulation (especially in the functions related to interferon regulation). BST2 protein can be activated by interferon and inhibit the synthesis of viral coat protein when cells are infected, thus playing an antiviral role (37). DDX58 can edit RIG-I protein, while RIG-I protein can detect viral nucleoprotein, and then activate interferon-stimulated genes (ISGs) (30). The above genes are all involved in the antiviral function. Some of the feature genes are also associated with negative immune regulation. For example, USP18k negatively regulates the interferon signaling pathway and dissociates ISG15 from binding to proteins of interest to inhibit the ubiquitination process (38). LAG3 acts as an immune checkpoint molecule to inhibit the activity of CD8+T cells (40). In addition, an article stated that ISG15 as a ubiquitin-like protein can be recognized and degraded by SARS-CoV-2, thereby repressing the ubiquitination activity (34). Since the process of protein ubiquitination modification is crucial to the regulation of the human immune system, the above process is possible to be involved in immune regulation (41). Therefore, we inferred that the 36 feature genes were highly correlated with SARS-CoV-2 infection. Further, we also performed GO enrichment analysis on the main subset of the PPI network of 36 feature genes, indicating the genes in the subset were largely enriched in the biological functions relating to response to virus and interferon signaling. From the results of enrichment analysis, it is deduced that the function of these feature genes was closely related to the immune regulatory response after SARS-CoV-2 infection.

Taken together with the above discussion, the 36 feature genes we selected were unique biomarkers in COVID-19 that could effectively distinguish the positive cases from the negative ones. Nevertheless, there were some limitations. We only predicted the candidate biomarkers for COVID-19, but did not study further mechanisms of them. Therefore, we plan to explore the role of some of these genes in the infected cells through a series of molecular as well as *in vitro* cellular functional experiments. For example, after SARS-CoV-2 infection, what would change in the LAG3 level; whether LAG3 inhibits the activity of CD8+T cells; if LAG3 affects the activity of CD8+T cells, which pathways would be affected and what is the significance of these effects.

## Data Availability Statement

The datasets presented in this study can be found in online repositories. The names of the repository/repositories and accession number(s) can be found in the article/Supplementary Material.

## Author Contributions

WC designed the study and reviewed and edited the manuscript. FX contributed to the literature research. YS and QZ collected the data. QY and MY analyzed and interpreted the data. YS wrote the initial draft of the manuscript. All authors read and approved the manuscript.

## Funding

The work was funded by Jiaxing Fight Novel Coronavirus Pneumonia Emergency Technology Attack Special Project in 2020 (No. 2020GZ30001), the Key Discipline of Jiaxing Respiratory Medicine Construction Project (No. 2019-zc-04), General Scientific Research Project of Education Department of Zhejiang Province (No. Y202043729), and Jiaxing Key Laboratory of Precision Treatment for Lung Cancer.

## Conflict of Interest

The authors declare that the research was conducted in the absence of any commercial or financial relationships that could be construed as a potential conflict of interest.

## Publisher's Note

All claims expressed in this article are solely those of the authors and do not necessarily represent those of their affiliated organizations, or those of the publisher, the editors and the reviewers. Any product that may be evaluated in this article, or claim that may be made by its manufacturer, is not guaranteed or endorsed by the publisher.

## Supplementary Material

Supplementary Table 1 Clinical characteristics of 234 samples downloaded from GEO database.

Supplementary Table 2 Top 50 feature genes selected *via* ReliefF, mRMR, and BorutaMCFS algorithm.
